# Type and Frequency of Misdiagnosis and Time Lag to Diagnosis in Patients with Chronic Progressive External Ophthalmoplegia

**DOI:** 10.18502/jovr.v19i3.13998

**Published:** 2024-09-16

**Authors:** Nasser Karimi, Hossein Ghahvehchian, Ali Keyhani, Amir Manavishad, Christopher J Compton, Jeremy D Clark, Nicole L West, Mohsen B Kashkouli

**Affiliations:** ^1^Department of Ophthalmology, Eye Research Center, The Five Senses Health Institute, Rassoul Akram Hospital, Iran University of Medical Sciences, Tehran, Iran; ^2^Department of Ophthalmology and Visual Sciences, University of Louisville School of Medicine, Louisville, Kentucky, USA; ^4^ Nasser Karimi: https://orcid.org/0000-0003-2045-6163; ^5^ Mohsen B Kashkouli: https://orcid.org/0000-0003-0347-5549

**Keywords:** Blepharoptosis, Chronic Progressive External Ophthalmoplegia, Diagnostic Errors, Myasthenia Gravis

## Abstract

**Purpose:**

Since ptosis is an early feature of chronic progressive external ophthalmoplegia (CPEO), patients are commonly misdiagnosed with other causes of ptosis. This study aims to report the type and frequency of misdiagnosis and time lag to diagnosis and the palpebral fissure transfer (PFT) procedure in patients with CPEO.

**Methods:**

This is a retrospective analysis of consecutive patients with CPEO who underwent PFT between 2006 and 2017. The data on previous diagnoses and treatments, age at definitive diagnosis of CPEO, and clinical manifestations were recorded. While the diagnosis of CPEO was based on clinical examination, 75% (24/32) of patients had undergone a confirmatory muscle biopsy and genetic tests.

**Results:**

There were 32 patients (19 females) with a mean age of 24.8 years (range, 13–36) at the final diagnosis and 34.1 years (range, 15–56) at the time of PFT. Also, 78% (25/32) of patients had been initially misdiagnosed with congenital ptosis (60%; 15/25) and ocular myasthenia gravis (OMG) (40%; 10/25). The majority of patients (20/32) had one to three previous eyelid surgical procedures, of which 90% (18/20) were performed before the definitive diagnosis of CPEO. The mean time lag from the first surgical procedure to CPEO diagnosis and PFT was 6.2 and 14.7 years, respectively.

**Conclusion:**

In a referral center, 78% of the patients with CPEO were initially misdiagnosed with congenital ptosis and OMG, and 56% of them underwent ptosis repair before the diagnosis. While the onset of the disease was in the first or second decades of life, diagnosis was delayed up to a mean age of 25 years. Reviewing early family photos and paying attention to other signs of CPEO could prevent misdiagnosis.

##  INTRODUCTION

Chronic progressive external ophthalmoplegia (CPEO) is a collective term for a genetically heterogeneous group of disorders, with an estimated prevalence of 1 in 30,000 of the general population with a slight female predominance (2.5:1).^[1]^ As the name implies, CPEO is characterized by slowly progressive, usually symmetric, ophthalmoplegia. Patients often (but not always) present with ptosis.^[1,2,3,4,5,6]^ As the downgaze is commonly restricted through the course of the disease, most patients report difficulty with reading. Visual impairment, poor blinking, exposure keratopathy, thinning of the outer retinal layers, and retinal nerve fiber layer defects are other ophthalmic findings.^[2,3,4]^ Systemic manifestations include generalized muscle weakness, dysphagia, and sensorineural hearing impairment. Elevated serum levels of alanine, lactate, creatine kinase, and fibroblast growth factor 21, and abnormal urine organic acid profiles are other findings in CPEO.^[1,5]^ Besides, CPEO in Kearns-Sayre syndrome is associated with pigmentary retinopathy and cardiac conduction abnormalities which typically present before the age of 20.^[7]^

Cardiac evaluation should be considered for all patients with CPEO to rule out conduction defects.^[8]^ CPEO is diagnosed based on the phenotype and the genotype. While blood and urine provide conveniently accessible DNA samples, some mitochondrial DNA variants are solely detectable in the high energy-demanding tissues, making muscle biopsy the diagnostic “gold standard”.^[6]^

Although there have been some case reports of misdiagnosis in individual patients with CPEO^[9]^-^[24]^ [Table 1], the present study is the largest case series addressing misdiagnosis in patients with CPEO. We have recently presented the results of the palpebral fissure transfer (PFT) procedure in 32 patients with CPEO,^[3]^ and now we aim to explore the characteristics of CPEO misdiagnosis in the same group of patients.

It is generally accepted that the treatment of ptosis in CPEO has a different goal (relief of chin up with the conservative opening of the visual axis) and technique (elevating the lower eyelid to minimize the palpebral fissure exposure) compared to congenital or involutional ptosis.^[3]^ As some CPEO patients may receive surgical treatment for ptosis prior to the correct diagnosis of the condition, another aim of this study is to assess the time lag to the diagnosis of CPEO and the time lag to the PFT (as the treatment of choice).

##  METHODS

This retrospective study was conducted on patients with CPEO who presented with ptosis or ptosis and diplopia to the senior author's (MBK) university and private practice from 2006 to 2017. The data on previous diagnoses and treatments, age at definitive diagnosis of CPEO, and clinical manifestations were extracted from the medical records.

Patients with CPEO were identified. While the diagnosis of CPEO was based on clinical examination,^[3]^ 75% (24/32) of patients had a confirmatory muscle biopsy and genetic tests, which were performed for another project.^[5]^ Thorough ocular and systemic examinations were done, and a consultation session was held with a cardiologist. Patients with other types of ptosis or follow-up of < 2 years were excluded. Data on age, sex, previous eyelid surgical operations, associated diseases, and eyelid characteristics were recorded. Binocular diplopia was categorized as present or absent. Primary outcomes were the frequency of misdiagnosis and prior surgical operations as well as time lag to diagnosis and PFT procedure. Patients' consent and IRB approval (IR.IUMS.REC.1401.187) were obtained, and the study followed the requirements of HIPAA and the Declaration of Helsinki.

Continuous variables were presented as mean ± standard deviation (SD) and categorical variables as absolute frequency and relative frequency (%).

##  RESULTS

Over an 11-year time period, 32 patients with COEP (19 females, 13 males, mean age 34.1 ± 11.2 years, range, 15–56) underwent bilateral upper eyelid ptosis and lower eyelid retraction repair, also known as the PFT procedure.^[3]^ The indication for PFT was to relieve the chin-up position. Table 2 summarizes the demographic data and perioperative characteristics of all these patients. Kearns-Sayre syndrome was detected in 12% (4/32) of the patients (mean age at diagnosis: 17.0 ± 2.8 years, range, 13–19).

Misdiagnosis was recorded in 78% (25/32) of the patients; specifically, it included congenital ptosis in 47% (15/32) and ocular myasthenia gravis (OMG) in 31% (10/32) of the patients. All patients with congenital ptosis (15/15) and three patients with OMG (3/10)—in other words, 56% of all patients—had a history of surgical operation. This group (*n* = 18) had their final CPEO diagnosis at a mean age of 25 ± 5.2 years (range, 18–35) and surgical operation at a mean age of 18.8 ± 3.9 years (range, 15–29), which translates into a time lag to a diagnosis of around six years.

**Figure 1:**
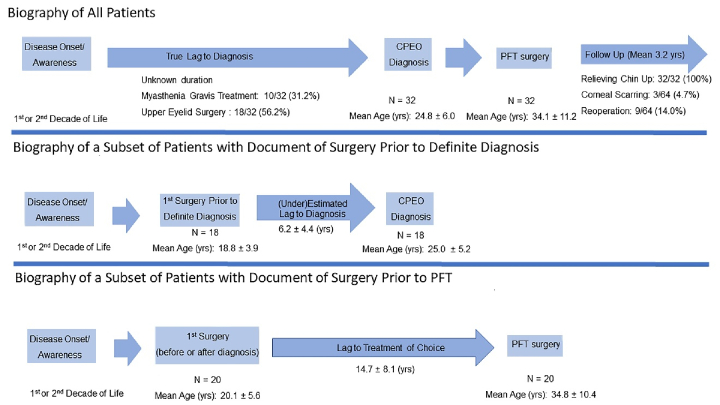
The time lag to diagnosis and treatment of choice (i.e., palpebral fissure transfer surgery) in patients with chronic progressive external ophthalmoplegia.

Of all patients, 62% (20/32) had a history of one to three eyelid surgeries prior to PFT. The first surgical operation in this group (*n* = 20) was carried out at a mean age of 14.7 ± 8.1 years (range, 4–30). The PFT, however, was performed at a mean age of 34.8 ± 10.4 years (range, 19–54) [Figure 1].

**Table 1:** Reported misdiagnosis in patients with chronic progressive external ophthalmoplegia and its related syndromes.


**Author and Date**	**Number**	**Sex and age**	**Initial diagnosis**	**Definitive diagnosis**
Sabella 2020^[9]^	2	Female,7.0	Pearson marrow pancreas syndrome	Kearns Sayre syndrome
Rajput 2018^[10]^	1	Female, 39.0	Myasthenia gravis	CPEO
Vahdani 2018^[11]^	1	Female, 64.0	Intracranial hypotension	CPEO
Bucelli 2016^[12]^	2	Male, 58.5	Other neurologic disorders	CPEO (without ptosis)
Dolores 2013^[13]^	1	Male, 64.0	Myasthenic crisis	CPEO
Holloman 2013^[14]^	1	Female, 11.0	Isolated growth failure	Kearns-Sayre syndrome
Ergul 2010^[15]^	1	Male, 11.0	Somatomedin C deficiency	Kearns-Sayre syndrome
Feddersen 2009^[16]^	1	Female, 21.0	Anorexia nervosa	Mitochondrial neurogastrointestinal encephalomyopathy
Surya 2008^[17]^	1	Male, 14.0	Kearns Sayre syndrome	Spinocerebellar ataxia type 7
Behbehani 2007^[18]^	1	Male, 25.0	Myasthenia gravis	CPEO
Ben Yaou 2006^[19]^	12	Female (58.4%), 47.0	Myasthenia gravis	Mitochondrial myopathies
Fijołek 2003^[20]^	1	Female, 30.0	Myasthenia gravis	Kearns-Sayre syndrome
Corrado 2002^[21]^	1	Female, 34.0	Polymyositis	Kearns-Sayre syndrome
van Domburg 1996^[22]^	6	Female (83%), 18.8	Hereditary sensory neuropathy	CPEO
Nørby 1994^[23]^	1	Female, 15.0	Psychosomatic disorder	Kearns-Sayre syndrome
Krendel 1987^[24]^	17	N/A	Myasthenia gravis	CPEO
	
	

##  DISCUSSION

In the present study, 78% (25/32) of the patients with CPEO had first received a misdiagnosis of congenital ptosis (15/25) and OMG (10/25). Notably, 56% (18/32) of the patients had undergone blepharoptosis surgery between 1 and 15 years (mean = 6.2 ± 4.4) before the final CPEO diagnosis. This time lag to diagnosis, averaging around six years, prevents patients from receiving timely information about the progressive nature of the disease before making any decision for surgery. This problem becomes even more critical if one considers that prior surgical operations for these individuals were performed at a mean age of 18.8 ± 3.9 years when adolescents often make serious plans for their future careers or family.

Most of these patients (83%; 15/18) had undergone levator resection surgery for congenital myogenic ptosis. Analyzing early family photos of these patients showed no evidence of congenital ptosis, which highlights how examining early family photos may reduce misdiagnosis in patients presenting with ptosis beyond childhood. Limitation of ocular motility, if any, could be a clue for diagnosing CPEO in patients presenting with ptosis for the first time. However, ptosis may precede motility limitations. Reducing the time lag to diagnosis can exert a considerable impact on attending premarital genetic counseling and choosing careers that are less dependent on visual function.

On the other hand, 31% (10/32) of the patients in our study were on OMG treatment. History taking and physical examination, although an essential first guide, cannot always distinguish between the OMG and CPEO. In contrast to the classic conception that CPEO rarely presents with diplopia because of symmetric ophthalmoparesis,^[25]^ 16% of the patients (5/32) in the present series were diplopic. Other studies have reported transient or constant diplopia in 28–62% of patients with CPEO.^[2,26,27]^ Associated diseases may provide diagnostic clues in favor of CPEO, but they are not frequently present. Kearns-Sayre syndrome was present in 12% (4/32) of patients in this series. Forced duction tests may identify restriction in the late stages of CPEO due to fibrotic changes in extraocular muscles,^[11]^ which is not a common finding in OMG. However, this test fails to distinguish early CPEO from OMG. Paraclinical tests are frequently utilized to differentiate CPEO from OMG. Thus, the diagnostic accuracy of single fiber electromyography (SFEMG) and ice pack test (IPT) were studied in 155 consecutive patients with clinical suspicion of OMG.^[28]^ The most common final diagnosis other than OMG was CPEO in 14% (22/155) of the patients, followed by intracranial lesions in 8% (12/155), cavernous sinus syndrome in 7% (11/155), and neuropathies in 5% (8/155). Of note, both tests were positive in 9% (2/22) of the patients with CPEO; in 18% (4/22) only SFEMG was positive, and in 14% (3/22) IPT alone was positive. Only in half of the patients with CPEO (11/22), both SFEMG and IPT were negative. This reveals that SFEMG- and/or IPT-positive cases may finally prove to be CPEO rather than OMG. The edrophonium test was positive in 100% (34/34) of the patients with OMG but none with CPEO. Notably, the edrophonium test may lead to an episode of cardiac block in patients with CPEO.^[29]^ There is also a report suggesting the diagnostic shortcoming of the neostigmine test in distinguishing CPEO from OMG. Le Forestier et al^[30]^ described 13 patients with a definitive diagnosis of CPEO (3 of their own patients plus 10 patients from the literature) who presented with ptosis and fatigable weakness and were initially evaluated at least by a neostigmine test (positive in five cases) or a repetitive nerve stimulation test (neuromuscular block in six cases). Studies suggest that serum acetylcholine receptor (AChR) antibody testing offers a specificity range of 95–100% for OMG;^[31]^ however, sensitivity may be as low as 36%.^[28]^ An atypical case of unilateral CPEO presenting with a false positive AChR antibody test highlighted the superiority of radioimmunoassay over enzyme-linked immunosorbent assay in the detection of AChR antibodies.^[10]^

These investigations seem to suggest that it may be difficult to accurately differentiate OMG and CPEO. Therefore, muscle biopsy (for detecting mitochondrial DNA mutations) should be considered in patients with suspected early OMG.^[6,32]^ Orbicularis oculi muscle biopsies, in case the patient is a candidate for ptosis surgery, may easily provide a muscle sample for this purpose.^[33]^ Patients with CPEO who are initially misdiagnosed with OMG are at risk of receiving unnecessary medications, including immunosuppressive drugs.

It is generally believed that the procedure of choice for patients with CPEO and ptosis should include elevation of the lower eyelid, in addition to upper eyelid ptosis correction.^[3]^ The rationale behind PFT is primarily to prevent excessive widening of the palpebral fissure in this vulnerable group of patients who generally lack corneal protective mechanisms (orbicularis function, Bell's phenomenon, and basal tear production).^[3]^ The time lag to PFT in the present series was around 15 years.

The present case series of 32 consecutive patients with CPEO provided an opportunity to assess diagnostic challenges in this rare clinical condition. Nevertheless, this study was prone to conceivable limitations of a retrospective study. Particularly, patients were not able to provide accurate data regarding the onset or awareness of their CPEO symptoms. Therefore, we decided to estimate the time lag to diagnosis based on exclusively objective data from those patients with medical records for eyelid surgery prior to the definitive diagnosis of CPEO. Although this method takes advantage of objective reliable data, it certainly underestimates the true time lag to diagnosis [Figure 1]. The lack of genetic testing for all patients in our series may give rise to some uncertainties about the diagnosis. Selection bias also needs to be considered, because it is likely that a group of patients with CPEO may have achieved acceptable results with ptosis surgery, even without or prior to correct diagnosis, and thus have not been referred to the subspecialty center. Hence, it is important not to extend the results of this study to the whole CPEO population. Rather, they should be interpreted within the context of a subspecialty referral center.

In summary, most patients with CPEO admitted to a subspecialty referral center were initially misdiagnosed with congenital ptosis and OMG, and almost half of them had ptosis repair before the correct diagnosis. The diagnosis was delayed up to the mean age of 25 years. Unfortunately, this delay occurred during late adolescence, a critical time when individuals make life-changing decisions such as marriage or their future careers. Reviewing early family photos and paying attention to other signs of CPEO could lead to a lower rate of misdiagnosis and an earlier diagnosis, hence avoiding unnecessary eyelid procedures and treatments.

### Financial Support and Sponsorship

None.

### Conflicts of Interest

Dr. Clark is a professional speaker for Horizon Therapeutics. No disclosure for the other authors.
